# Potential for Algorithmic Bias in Clinical Decision Instrument Development

**DOI:** 10.1038/s41746-025-02119-7

**Published:** 2025-12-10

**Authors:** Jed Keenan Obra, Chandan Singh, Kenshata Watkins, Jean Feng, Ziad Obermeyer, Aaron Kornblith

**Affiliations:** 1 University of California, Berkeley, Berkeley, CA USA; 2 University of California, San Francisco, San Francisco, CA USA; 3 Microsoft Research, Seattle, WA USA

**Keywords:** Predictive medicine, Diagnosis, Outcomes research, Clinical trial design

## Abstract

Clinical decision instruments (CDIs) face an equity dilemma. They reduce disparities in patient care through data-driven standardization of best practices. However, this standardization may perpetuate bias and inequality within healthcare systems. We perform a quantitative, systematic review to characterize four potential sources of bias in the development of 690 CDIs. We find evidence for potential algorithmic bias in CDI development through various analyses: self-reported participant demographics are skewed—e.g. 73% of participants are White, 55% are male; investigator teams are geographically skewed—e.g. 52% in North America, 31% in Europe; CDIs use predictor variables that may be prone to bias—e.g. 1.9% (13/690) of CDIs use *Race and Ethnicity*; outcome definitions may introduce bias—e.g. 26% (177/690) of CDIs involve follow-up, which may skew representation based on socioeconomic status. As CDIs become increasingly prominent in medicine, we recommend that these factors are considered during development and clearly conveyed to clinicians.

## Introduction

Clinical decision instruments (CDIs) are tools used in healthcare to assist clinicians in diagnosing conditions, predicting patient outcomes, or guiding treatment decisions based on clinical data. These instruments are widely used with the aim of improving patient care through data-driven standardization^1,2^. While CDIs generally enhance clinical care, this standardization may inadvertently perpetuate inequality within healthcare systems^3–6^. As data-driven CDIs become more prominent in medicine, care must be taken to ensure that they do not perpetuate algorithmic bias, which we define as less accurate or effective guidance for patients across different socio-economic, racial, ethnic, or other demographic groups^7^. Algorithmic bias arises from various factors that influence the CDI algorithm, potentially affecting the generalizability of the CDI toward specific demographic groups^4,7^. Throughout this manuscript, we use “algorithmic bias” to discuss upstream data or design choices that *may* degrade model performance for a sub-population; we do not claim these factors are themselves biased until differential performance is demonstrated. Where performance bias is documented, we label it “observed bias”, and we restrict the term “implicit bias” to human clinical decision-makers, following social-psychology conventions^8^.

Various factors can introduce algorithmic bias during the CDI development process. Perhaps most importantly, failing to enroll diverse populations in development cohorts can lead to inaccuracies, disproportionately affecting minority groups^9^. Beyond enrollment, bias can arise from the predictor variables included in the CDI (e.g. *Race*)^9–11^ or in the definition of the outcome itself, which may skew the representation of different groups^12,13^. Similarly, the regional affiliations and demographics of investigator teams may influence the perspectives and biases embedded in these instruments^14^.

Here, we take a closer look at these disparate factors contributing to CDI bias. Specifically, we analyze the CDIs in MDCalc^15^, an online tool that hosts hundreds of widely used CDIs. We find potential for bias introduced through four factors: patient cohort demographics, predictor variables, outcome definitions, and author demographics. Going forward, we recommend that these factors (in addition to standard best practices^16^) are conveyed clearly to clinicians, e.g. in a model card^17^. Moreover, the CDI development process for new CDIs should carefully consider these sources of algorithmic bias in order to design equitable CDIs that work for diverse populations.

## Results

### Overview and characteristics of analyzed CDIs

**Fig. 1:**
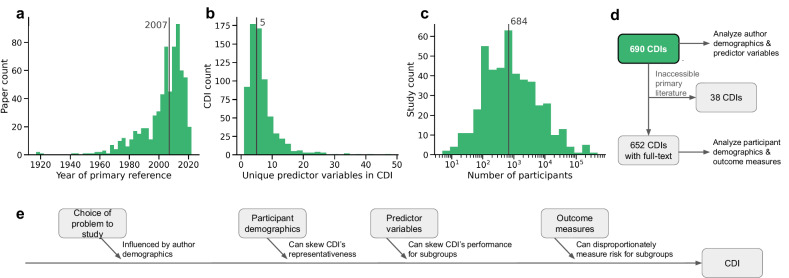
Overview of analyzed CDIs and key characteristics. The CDIs demonstrate diversity across several characteristics, including publication year **a**, number of predictor variables **b**, and size of the development cohort **c**. Vertical lines represent the median values for each histogram. **d** A total of 690 CDIs were analyzed; 652 of these studies yield a full-text primary reference. **e** For each CDI, we analyze four factors that may introduce bias during CDI development.

We conducted a comprehensive analysis of CDIs by collecting key data for all CDIs available on the MDCalc website as of March 17, 2023. This process yielded a dataset of 690 CDIs, categorized by disease, system, purpose, chief complaint, and specialty according to MDCalc’s classification (Table 1), each with their own individual development study. The CDIs included in the analysis exhibited diversity across several relevant characteristics. Most CDIs are fairly recent (median publication year 2017; see Fig. 1a), although the earliest publication in our data dates back to 1917. The number of new CDIs added each year has generally increased over time, with a recent decline corresponding to CDIs awaiting validation for inclusion in MDCalc. The number of predictor variables in the CDIs varied, with a median of 5 and a maximum of 48 (Fig. 1b). Additionally, the size of the development cohorts used to develop the CDIs varied widely (minimum 5, median 853, maximum 490, 768; see Fig. 1c).

**Table 1:** Categorizing CDIs by MDCalc’s classification system

Disease	#	System	#	Purpose	#	Chief complaint	#	Specialty	#
Trauma	64	Gastrointestinal	129	Prognosis	345	Shortness of Breath	146	Internal Medicine	457
Cancer	62	Cardiac	128	Diagnosis	277	Abdominal Pain	115	Hospitalist Medicine	374
Stroke/TIA	55	Neurologic	126	Calculation	93	Chest Pain	107	Emergency Medicine	332
COVID-19	54	Respiratory	102	Treatment	81	Weakness	104	Critical Care	276
Myocardial Infarction	44	Hematologic	72	Rule Out	41	Fever	80	Family Practice	248
Disorders of Gut-Brain Interaction	42	Oncologic	67			AMS	63	Primary Care	176
Pulmonary Embolism	30	Endocrine and Metabolic	49			Vomiting	62	Hematology and Oncology	129
Heart Failure	28	Renal	45			Injury/Trauma	60	Gastroenterology	124
Respiratory Distress	27	Musculoskeletal	42			Fatigue	53	Cardiology	123
Cirrhosis	26	Psychiatric	42			Weight Loss/Gain	52	Neurology	111
Hepatitis	26	Infectious	36			Bleeding	50	Surgery (General)	105
Pneumonia	25	Immunologic	25			Headache	48	Pulmonology	96
Renal Failure	25	Urinary	20			Pain	47	Pediatrics	77
Diabetes Mellitus	25	Vascular	20			Anxious	47	Geriatrics	65
Pneumonia	25	Rheumatologic	20			Dyspnea	46	Surgery (Trauma)	55

690 CDIs classified by disease, system, purpose, chief complaint, and specialty; only the top 10 most frequent values for each column are shown. CDIs may be labeled with multiple classes for a given categorization, e.g. a CDI can be categorized both under *Disease: Respiratory Distress* and *Disease: Pneumonia*. *TIA Transient ischemic attack, AMS Altered mental status*.

To further analyze these 690 CDIs, we collected the full-text of the primary study whenever it is available, resulting in 652 CDIs with primary text (Fig. 1d); the 38 CDIs without accessible primary development literature are shown in Supplementary Table 7. In what follows, we analyze four factors that may introduce bias during CDI development (Fig. 1e).

### Participant demographics

**Fig. 2:**
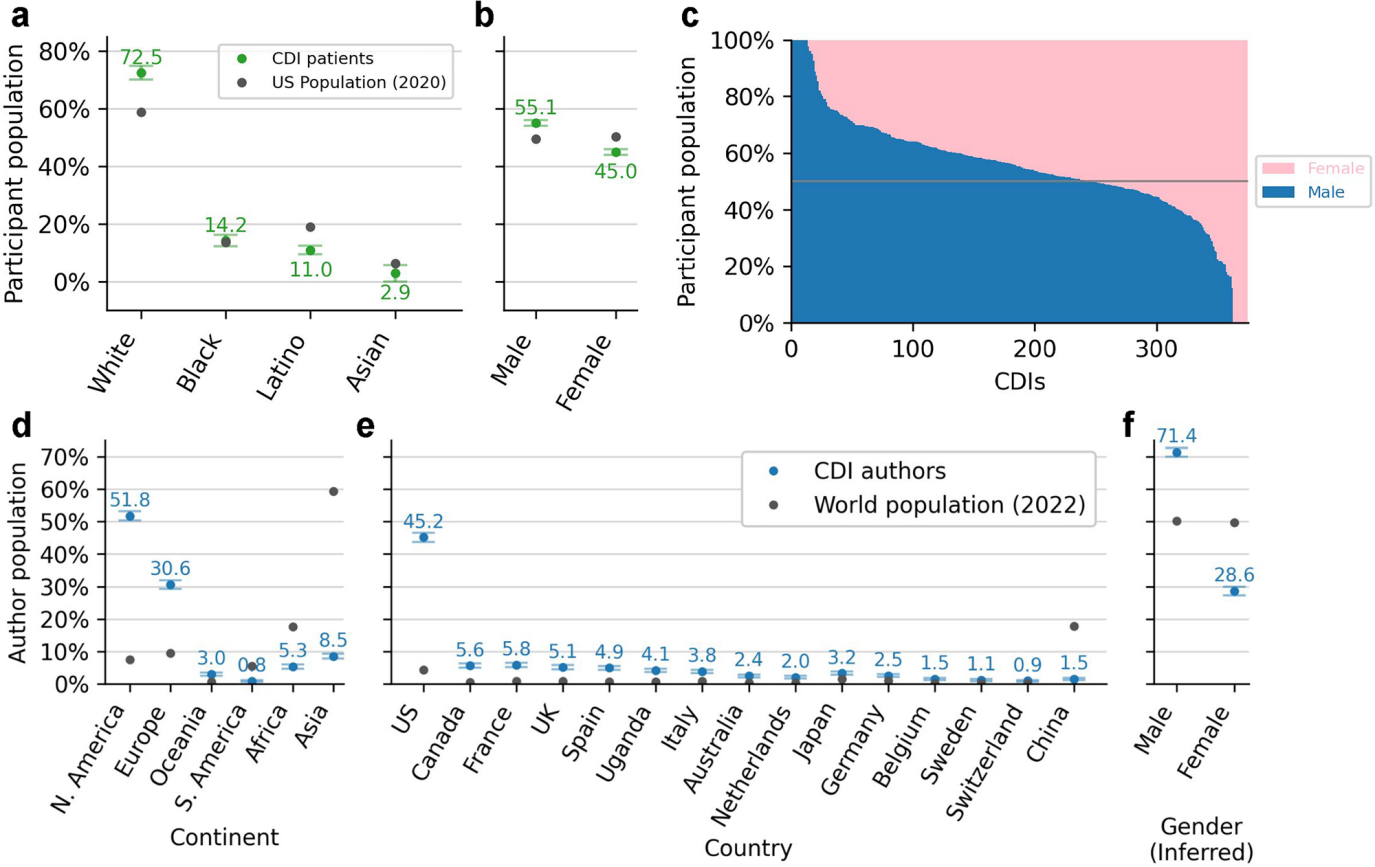
Demographics (self-reported) of CDI development participants and study authors. **a** Race and Ethnicity distribution of CDI development cohort participants compared to the US population across four racial categories; White, Black (or African American), Latino (or Hispanic), and Asian (or Pacific Islander). **b** Sex distribution and **c** sex breakdown for the participant population of each CDI. 242 of the 374 CDIs (65%) had a higher proportion of male participants than female participants. Author population distribution by **d** continent, **e** country, and **f** inferred author gender via validated ChatGPT name-gender algorithm; 99% accuracy on 81 manual checks. All values are sorted by the difference between the CDI author population and world population. Error values plot the median over CDI primary studies (to avoid outlier effects) and error bars show standard error of the mean. Each country not shown accounts for less than 1% of CDI authors.

We evaluated the demographics of participant cohorts used in CDI development to assess potential algorithmic bias. Figure 2a compares the racial and ethnic distributions of CDI participant cohorts with the U.S. population in 2020^18^. Note that different CDIs may be suitable for different populations, but as these are not marked consistently in the manuscripts or on MDCalc, we compare participant data to US population demographics. CDI cohorts significantly over-represent White-only participants (*p* = 0.012) and significantly under-represent Latino populations (*p* = 0.002*)*. All *p*-values were calculated using 1-sample t-test with Benjamini-Hochberg false discovery rate correction^19^.

We further examined the sex distribution of participants in CDI development cohorts (Fig. 2b) and found a significant over–representation of male participants compared to female participants (*p* < 10^–5^). As shown in Fig. 2c, 65% (242/374) of CDIs had a higher proportion of male participants than female participants. Additionally, 25 CDIs included participants of only one sex: 13 comprised exclusively male participants, and 12 comprised exclusively female participants. While most of these single-sex cohorts were for sex-specific conditions (e.g. testicular torsion^20^ or ovarian cancer^21^), some were not (e.g. HAM-D scale for depression^22^); see Supplementary Table 4 for a list of all CDIs with single-sex cohorts.

### Author demographics

In a secondary analysis, we quantified potential biases in the author population of primary CDI studies. Specifically, we identified 3,819 author names and 1,234 author affiliations across 690 CDI studies and compared their demographics to the worldwide demographic distributions^23^. Figure 2d, e shows the distribution of author affiliations by continent or country. Authors are overwhelmingly in North America (52%) or Europe (31%) and nearly half of all authors are in the US (45%); all these discrepancies are significantly greater than the world population baseline (*p* < 10^-5^). Figure 2f further shows that sex distributions are skewed male even more than the participant population (71%), drastically more than worldwide demographics^24^ (*p* < 10^-5^). These disparities are consistent with author demographics trends seen across medical publications beyond CDIs^25,26^. The link between author demographics and bias in a resulting CDI is weaker than other sources of potential bias, such as participant demographics. Nevertheless, while author demographics may not directly introduce bias, they may influence factors such as which outcomes are studied and which patient population is enrolled.

### Predictor Variables

We further investigated predictor variables that may be susceptible to introducing bias within CDIs, i.e. unintentionally providing less accurate or effective guidance for patients in particular subgroups. However, note that any of these predictor variables may not introduce bias, or may even correct for unintentional biases if used carefully^27^.

**Fig. 3:**
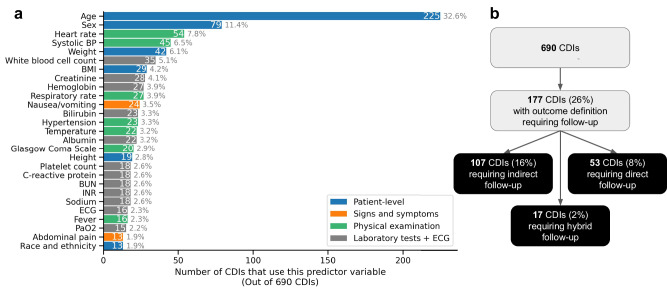
Frequency of predictor variables in MDCalc CDIs. **a** Colors represent different categories of predictor variables dependent on type of collection. Blue represents participant-level predictor variables. Orange represents signs and symptoms upon participant presentation. Green represents physical examination findings. Gray represents laboratory tests including ECG. **b** Flowchart of CDIs categorized by follow-up. BP Blood pressure, BMI Body mass index, BUN Blood urea nitrogen, INR International normalized ratio blood test, ECG electro cardiogram. Variables appearing in less than 13 CDIs are not shown.

Figure 3a shows the most frequently used predictor variables in the MDCalc CDIs. We identify three common predictor variables which may be susceptible to introducing bias. First, *Abdominal pain* (present in 1.9% of CDIs) may be susceptible to clinical misinterpretation when severity is coded by a clinician rather than patient. Second, *Race and Ethnicity* (present in 1.9% of CDIs) may inadvertently skew predictions for racial groups, depending on its usage during CDI development. Race/ethnicity variables can encode structural inequities (e.g., differential access to diagnostic work-ups) that propagate into model parameters. Third, *Family History* (present in 1.4% of CDIs) may indirectly be influenced by past familial medical knowledge, leading to differing impacts. Family history items can penalize recent immigrants or patients lacking documented pedigree information by reducing the number of risk factors input in calculations that influence clinical decision-making for screening or treatment^27^. We list the CDIs that explicitly use these predictor variables in Supplementary Tables 1 and 2. The most common variable is Age (present in 32.6% of CDIs); we find that many of the most frequent predictor variables, e.g. *Sex*, tend to not contribute significant influence to the CDI to determine its outcome measure (see Supplementary Fig. 1). Quantifying this frequency helps identify variables that may be more important to study when understanding potential bias introduced by the variables within the CDI algorithm.

### Outcome measures

Finally, we investigated the outcome definition itself as a source of CDI bias. While there are many potential factors of the outcome definition, we specifically focused on the presence of *follow-up* in the outcome definition. Access to follow-up can vary by subgroup due to socioeconomic constraints^28^, which can lead to differential verification bias. For example, the Canadian CT Head Rule^29^, excluded patient data from analysis if investigators could not reach patients for follow-up. We define follow-up as a continuous recording of health data following an initial care visit. Active ascertainment of an outcome describes telephone or in-person visits, while passive ascertainment of an outcome defines surveillance of electronic records or registries. This factor potentially introduces bias, as wealthier participants are more likely to receive follow-up and meet the criteria for the outcome definition. We manually identified 177 CDIs (26%) that involve follow-up as part of the primary outcome definition (Fig. 3b). Of these, 53 require active follow-up and 17 require hybrid follow-up (together 10% of CDIs), both categories that are more prone to bias than passive follow-up. See Supplementary Table 6 for a full list of these CDIs involving follow-up.

## Discussion

Our study analyzes 690 MDCalc CDIs and provides the first quantitative review of “potential bias” in CDIs. The work here adds to a growing literature that finds broad evidence for bias in data-driven clinical algorithms and recommends improvements in best practices for CDI development^2,30–34^. We now briefly discuss the four factors related to CDI development we analyzed along with concrete takeaways for developers and users of CDIs.

The over-representation of male participants in developing CDIs has various explanations and may raise concerns about the broader applicability of particular CDIs. The male skew may be rooted in past Western regulatory policies advising the exclusion of women of childbearing potential from studies^35^. This skew may have persisted past these policies, as investigators sought to maintain consistency with prior male-only clinical trials^36,37^. Today, quantifying this skew can be challenging, as literature often fails to report results by sex and other demographics, even after guidance and mandates from the NIH^38,39^. Going beyond enrollment, many studies may specifically focus on male-specific health issues, e.g. testicular torsion and prostate cancer. Therefore, we recommend that bias mitigation starts as early as designing the enrollment process for participants to optimize the generalizability of the CDI.

The demographic and geographic skews we observed in CDI development (Fig. 2) can harm the global generalizability of CDIs. Specifically, CDIs are largely developed in Western countries and often used in non-Western countries with differing populations and disease profiles^40^. For example, the Kruis Score for diagnosing irritable bowel syndrome (IBS) enrolled a development cohort that overrepresented women, which is consistent with the higher prevalence of IBS in women in Western countries^41^. However, IBS prevalence has been shown to be equally common between males and females in Asia, highlighting how risk factors can be limited by the regional demographics of a study population^42^. To mitigate the effects of these demographic skews, we recommend that CDI developers should disclose awareness of risk factors for disease in their methodology for enrolling participants to highlight the limited generalizability of their instrument. Moreover, to reduce potential harm in CDI usage, validation studies should be conducted to test CDIs on underrepresented populations of the development cohort to assess generalizability prior to being officially approved for use in clinical settings.

The inclusion of predictor variables which are susceptible to bias is nuanced, and therefore should be treated with caution. For example, *Race* as a predictor variable has been subject to a great deal of scrutiny^9,10,43^, even leading to modern revisions for some instruments without Race, e.g. in eGFR measurement for kidney function^44^ or prediction of vaginal birth after cesarean delivery^45^. However, these same race adjustments, if used correctly in a CDI, may help correct for racial disparities in data quality^27^. CDI developers and clinicians should be aware of the potential bias introduced through race variables (in line with MDCalc’s Statement on Race^46^). Going beyond Race, CDIs should communicate to clinicians the biases that particular predictor variables may be susceptible to introducing, e.g. clinician perceptions of pain. Likewise, when external validation reveals worse performance for an underrepresented group, developers may consider decision pathways when facing a trade-off between adding a sensitive predictor (e.g., self-identified race, ethnicity, and sex) and redesigning the model to remove upstream biases^47,48^.

Outcome measures are a prevalent and often understudied source of bias in clinical algorithms^28^. We limited our analysis to studying *follow-up* (both active and passive) as an alternative reference standard for the primary outcome. However, there may be systemic implicit bias related to follow-up procedures that unintentionally limit the types of participants to those who can comply with follow–up methodology for the primary outcome. Active follow-up poses concern for bias as in-person visits and telephone communication can be limited by transportation and phone accessibility, low-income status, physical disability, and language barriers. To deal with this, some analyzed CDI development studies needed to either exclude patient data (e.g. HEART Score^49^) or input assumed data (e.g. Modified NIH Stroke Scale/Score^50^) to retain appropriate presence within the study dataset for patients who lack follow-up data. Passive follow-up raises different concerns with the wide usage of research databases within CDI development. While these databases often hold large sets of patient information, they are subject to data limitations such as missing values, small effective sample sizes and measurement error, each of which can *induce* bias if not handled appropriately^30^. Switching clinicians and areas of living may lead to their data being stored in multiple electronic databases that do not communicate with each other. CDI developers must remain aware of data fragmentation among patients with transient living situations.

Our study has several important limitations. First, we only analyzed primary literature within MDCalc, which may not provide a perfectly accurate representation of the international landscape of CDI development and usage. Moreover, we perform analysis using metadata but do not look directly at underlying data and algorithms used to develop CDIs. It is possible that active or passive follow-up was included in CDI studies but were not discussed or specified within the study methods. We compared CDI participant data to US population data because it was difficult to get worldwide data on racial and ethnic demographics. However, it is clear that disparities from the representation of minority groups are exacerbated when comparing CDI populations to worldwide populations (e.g. White over-representation and Asian under-representation). Bias can manifest itself in many ways beyond the components of CDI development that we study here including the validation and formulation phases. Future research should explore other potential signals of bias such as subjective clinician assessments and the use of costly diagnostic tests in CDI development. This study was not registered in an open-access database (i.e. PROSPERO or OSF) during this research.

In conclusion, we found CDIs may be biased at the patient, author, predictor, and outcome level. We recommend that algorithmic bias is taken into consideration by all parties involved in CDI usage: (i) investigators developing and validating CDIs, (ii) services implementing CDIs, e.g. MDCalc, and (iii) clinicians using CDIs. Investigators creating CDIs must consider and report numerous factors, including the demographics of their enrollment cohort and their predictor/outcome variables. To promote transparency and equitable use of CDIs, we recommend that online platforms and journals adopt standardized reporting frameworks. One such framework is the ‘model card,’ originally developed in machine learning to summarize a model’s purpose, development context, performance, and limitations^17^. We propose that a similar structure be used for CDIs, augmented with a ‘bias analysis’ that highlights participant cohort demographics, external validation data, and any known disparities in performance across patient subgroups. Such tools would allow clinicians to more easily evaluate the generalizability and limitations of a CDI before applying it in practice. While CDIs hold great potential for enhancing patient care through data-driven standardization, our findings underscore the need for careful consideration of bias in their development. By addressing these biases, we can work towards the development of more equitable and effective CDIs, ultimately contributing to more inclusive healthcare systems. Future research should continue to explore and quantify potential roots of bias in CDI development, and work towards developing strategies to mitigate these biases.

## Methods

### Inclusion criteria for primary studies on CDIs

To identify signals of bias within CDI development, we analyzed studies, labeled “Original/Primary Reference” on MDCalc, detailing the procedures by which CDI developers used to create their instruments and reviewed to ensure an instrument was developed. Using web data extraction, we collected studies from the MDCalc website (MDCalc.com, July 26, 2023) under the *Evidence* and *Original/Primary Reference* sections. We then manually searched and retrieved CDI primary literature using PubMed with the same title, authorship, and Digital Object Identifier (DOI). Primary development literature describes the earliest peer-reviewed report, as determined through expert review and by MDCalc contributors, that (i) describes development of the CDI on an original patient cohort and (ii) presents sufficient detail to reproduce the score. These development studies would include predictor and outcome variables upon description of the development of the instrument. When key metadata were split across multiple papers we extracted information from all and cited the index paper. For studies that were not available on PubMed, we used the Bioscience, Natural Resources & Public Health Library of University of California, Berkeley to find full-texts of the primary references. Two team members reviewed each text to confirm primary literature of CDI. To prevent repeat measurement of variables and factors used to develop each algorithm, one development study was evaluated for each CDI, and validation studies were not included. Validation studies describe research articles published after the earliest peer-reviewed report and that analyze the CDI beyond the development cohort. We excluded primary development literature that (i) was not written in English, (ii) exclusively described a validation study, (iii) used a textbook reference or established guideline (DSM-V) was used instead of a development research study, or (iv) was not available in library databases. Due to the heterogeneity of methodology between CDIs, no formal critical appraisal using risk of bias tools took place. The observation of CDI development methods to record participant and investigator characteristics, predictor variables, and outcome variables to assess bias classifies this study as a systematic review^51^. Methodological standards were verified with the Prisma 2020 checklist (Supplementary Table 8) for systematic reviews of studies^52^.

### Characterizing participant demographics in primary studies

To identify subject characteristics that may be overrepresented, we collected participant count, biological sex, and racial/ethnic demographic info of participant cohorts. Keywords include, *Asian*, *Caucasian*, *Hispanic*, *Latino*, *African*, etc. To verify the accuracy of web extraction data and missing information, we verified participant demographic data by direct examination of the article. The keywords *male*, *female*, *sex*, and *gender* were manually searched to identify count and biological sex. Primary literature uses both *sex* and *gender* terminology to describe biological sex of their participant cohorts as well as *race* and/or *ethnicity* to describe groups of participants connected by common descent. Aligned with the Journal of the American Medical Association (JAMA)^53,54^, we solely use the term *sex* to report biological factors, *gender* to report gender identity, and *race and ethnicity* to describe major groupings assigned in the literature while recognizing the origin of these terms as social constructs differentiating common descent and cultural identity.

### Characterizing investigator demographics in primary studies

To study secondary factors that may impact bias, authors, affiliations, and year of publication for each primary reference CDI study were identified from Pubmed. We excluded 125 CDIs in which the author’s name could not be analyzed (e.g. only initials available on authorship creditation). We used ChatGPT (GPT-3.5-Turbo, accessed through OpenAI’s python interface^55^) to automatically infer the gender identity of each author and to identify the country name corresponding to their affiliation. Specifically, we prompt ChatGPT with the input, *Return whether the name ‘{name}’ is more common for a male or a female. Answer with one word, ‘Male’ or ‘Female’*. To ensure that ChatGPT gender categorizations are reliable, the ChatGPT output was validated by matching the gender pronouns given on an author’s homepage. We searched 150 randomly selected author name/affiliation pairs (75 predicted as male, and 75 predicted as female) and 81 ground truth author gender were available from author homepages. 80 of 81 (99%) author genders were correctly identified by ChatGPT.

### Predictor variables and categorization

We used curated information from MDCalc contributors to identify CDI predictor variables and classify CDI by disease, system, purpose, chief complaint, and specialty. Predictor variable names used in each CDI were also identified from each CDI’s MDCalc page. The predictor variables were grouped by mode of collection. Age, sex, weight, BMI, and race and ethnicity were considered *patient-level* variables. Nausea/vomiting, abdominal pain, fever, etc. were considered *signs and symptoms* variables. Vital signs and Glasgow Coma Scale score were considered *physical examination findings*.

Due to variability in predictor variable nomenclature between CDIs measuring identical values (e.g. “Systolic Blood Pressure” and “sBP”), naming modifications were applied upon data extraction to rename these predictor variables to match each other (e.g. “Systolic Blood Pressure” → Systolic BP; “sBP” → Systolic BP). All nomenclature modifications are displayed in Supplementary Table 3. We defined other variables that may cause bias, such as those related to pain (e.g. Abdominal Pain) and family history. Family history is defined by the presence of chronic illness within the participant’s family resulting in a factor within the CDI’s algorithm calculation. CDIs with biased predictor variables, such as family history and race and ethnicity, were grouped and shown in Supplementary Tables 1 and 2. We additionally classify if these variables were used as statistical predictors influencing likelihood of disease onset, eligibility criteria describing inclusion criteria for CDI development cohort enrollment, or risk modifiers influencing severity of disease.

### Outcome variables

Outcome definitions and measures were manually identified from the text of each study. Outcome definitions describe the intended purpose or measurement of the CDI. These goals aim to include recommendations of medical treatment, diagnostic measure, or prognosis upon participant diagnosis. Outcome measures are defined as the benchmark measures used during the CDI’s development to test CDI internal validity. Recorded outcome measures include gold standard measures, previously validated measures from past literature, and/or reference measures. Outcome measures were not recorded if the primary article was a review, meta-analyses without explicit outcome measure description, or a descriptive article.

Determining inclusion of follow-up methodology as part of the outcome definition was conducted by assessing CDI study methodology for the implementation of recording data between at least 2 time points. Keywords assessed in methodology include *follow up, follow, database, dataset, phone*, etc. Active follow-up was defined by in-person or telephone follow up specifically for participation within study outcome. Passive follow-up was defined by researcher usage of databases or registries to identify patient data or health outcomes. Prospective observational studies were determined to be passive follow-up. Meta-analyses were excluded, whilst relevant primary studies on CDI development were included. Additionally, follow-up was included only when outcome definition could not be adjudicated without a second time-point (e.g., 30-day mortality). At least 2 study team members separately reviewed each outcome measure to determine if follow-up was involved. A third study team member reconciled any conflicting determination of follow-up.

## Supplementary information

Supplementary Information

## Data Availability

All data for reproducing the results in this manuscript is made publicly available through MDCalc and processed data is made available at https://github.com/csinva/clinical-rule-development. All code for reproducing the results in this manuscript is made publicly available at https://github.com/csinva/clinical-rule-development.

## References

[1] Reilly, B. M. & Evans, A. T. Translating clinical research into clinical practice: impact of using prediction rules to make decisions. *Ann. Intern. Med.* **144**, 201–209 (2006). 16461965 10.7326/0003-4819-144-3-200602070-00009

[2] Stiell, I. G. & Bennett, C. Implementation of clinical decision rules in the emergency department. *Acad. Emerg. Med* **14**, 955–959 (2007). 17923717 10.1197/j.aem.2007.06.039

[3] Paulus, J. K. & Kent, D. M. Predictably unequal: understanding and addressing concerns that algorithmic clinical prediction may increase health disparities. *Npj Digit. Med.* **3**, 1–8 (2020). 32821854 10.1038/s41746-020-0304-9 PMC7393367

[4] Obermeyer, Z., Powers, B., Vogeli, C. & Mullainathan, S. Dissecting racial bias in an algorithm used to manage the health of populations. *Science* **366**, 447–453 (2019). 31649194 10.1126/science.aax2342

[5] Mauro, M. et al. A scoping review of guidelines for the use of race, ethnicity, and ancestry reveals widespread consensus but also points of ongoing disagreement. *Am. J. Hum. Genet.* **109**, 2110 (2022). 36400022 10.1016/j.ajhg.2022.11.001 PMC9808506

[6] Callier, S. L. The Use of Racial Categories in Precision Medicine Research. *Ethn. Dis.* **29**, 651 (2019). 31889770 10.18865/ed.29.S3.651 PMC6919973

[7] Obermeyer, Z. et al. *Algorithmic Bias Playbook*. (Chicago Booth, The Center for Applied Artificial Intelligence, 2021).

[8] Shah, H. S. & Bohlen, J. Implicit Bias. in *StatPearls* (StatPearls Publishing, Treasure Island (FL), 2025). 36944001

[9] Hidden in Plain Sight — Reconsidering the Use of Race Correction in Clinical Algorithms | New England Journal of Medicine. https://www-nejm-org.libproxy.berkeley.edu/doi/full/10.1056/NEJMms2004740. 10.1056/NEJMms2004740 32853499

[10] Perez-Rodriguez, J. & de la Fuente, A. Now is the Time for a Postracial Medicine: Biomedical Research, the National Institutes of Health, and the Perpetuation of Scientific Racism. *Am. J. Bioeth. AJOB* **17**, 36–47 (2017). 28829268 10.1080/15265161.2017.1353165

[11] Ozanne, E. M. et al. Bias in the Reporting of Family History: Implications for Clinical Care. *J. Genet. Couns.* **21**, 547–556 (2012). 22237666 10.1007/s10897-011-9470-x

[12] Howe, C. J., Cole, S. R., Lau, B., Napravnik, S. & Eron, J. J. Selection Bias Due to Loss to Follow Up in Cohort Studies. *Epidemiol. Camb. Mass* **27**, 91–97 (2016). 10.1097/EDE.0000000000000409 PMC5008911 26484424

[13] Syed, S. T., Gerber, B. S. & Sharp, L. K. Traveling towards disease: transportation barriers to health care access. *J. Community Health* **38**, 976–993 (2013). 23543372 10.1007/s10900-013-9681-1 PMC4265215

[14] Love, H. B., Stephens, A., Fosdick, B. K., Tofany, E. & Fisher, E. R. The impact of gender diversity on scientific research teams: a need to broaden and accelerate future research. *Humanit. Soc. Sci. Commun.* **9**, 1–12 (2022).

[15] MDCalc - Medical calculators, equations, scores, and guidelines. *MDCalc* https://www.mdcalc.com/.

[16] Collins, G. S., Reitsma, J. B., Altman, D. G. & Moons, K. G. Transparent reporting of a multivariable prediction model for individual prognosis or diagnosis (TRIPOD): the TRIPOD Statement. *BMC Med* **13**, 1 (2015). 25563062 10.1186/s12916-014-0241-z PMC4284921

[17] Sendak, M. P., Gao, M., Brajer, N. & Balu, S. Presenting machine learning model information to clinical end users with model facts labels. *NPJ Digit. Med.* **3**, 41 (2020). 32219182 10.1038/s41746-020-0253-3 PMC7090057

[18] Bureau, U. C. Census.gov | U.S. Census Bureau Homepage. *Census.gov* https://www.census.gov/en.html.

[19] Benjamini, Y. & Hochberg, Y. Controlling the False Discovery Rate: A Practical and Powerful Approach to Multiple Testing. *J. R. Stat. Soc. Ser. B Methodol.* **57**, 289–300 (1995).

[20] Barbosa, J. A. et al. Development and initial validation of a scoring system to diagnose testicular torsion in children. *J. Urol.* **189**, 1859–1864 (2013). 23103800 10.1016/j.juro.2012.10.056

[21] Jacobs, I. et al. A risk of malignancy index incorporating CA 125, ultrasound and menopausal status for the accurate preoperative diagnosis of ovarian cancer. *Br. J. Obstet. Gynaecol.* **97**, 922–929 (1990). 2223684 10.1111/j.1471-0528.1990.tb02448.x

[22] Hamilton, M. A rating scale for depression. *J. Neurol. Neurosurg. Psychiatry* **23**, 56–62 (1960). 14399272 10.1136/jnnp.23.1.56 PMC495331

[23] World Population Prospects 2022: Summary of Results | Population Division. https://www.un.org/development/desa/pd/content/World-Population-Prospects-2022.

[24] The CIA World Factbook 2021-2022. *Skyhorse Publishing* https://www.skyhorsepublishing.com/9781510763814/the-cia-world-factbook-2021-2022.

[25] Charpignon, M.-L. et al. Does diversity beget diversity? A scientometric analysis of over 150,000 studies and 49,000 authors published in high-impact medical journals between 2007 and 2022. *MedRxiv Prepr. Serv. Health Sci*. 2024.03.21.24304695 10.1101/2024.03.21.24304695 (2024).

[26] Saloojee, H. & Pettifor, J. M. Maximizing Access and Minimizing Barriers to Research in Low- and Middle-Income Countries: Open Access and Health Equity. *Calcif. Tissue Int.* **114**, 83 (2023). 37962622 10.1007/s00223-023-01151-7 PMC10803444

[27] Zink, A., Obermeyer, Z. & Pierson, E. Race adjustments in clinical algorithms can help correct for racial disparities in data quality. *Proc. Natl. Acad. Sci. USA* **121**, e2402267121 (2024). 39136986 10.1073/pnas.2402267121 PMC11348319

[28] Yan, M., Pencina, M. J., Boulware, L. E. & Goldstein, B. A. Observability and its impact on differential bias for clinical prediction models. *J. Am. Med. Inform. Assoc.* **29**, 937–943 (2022). 35211742 10.1093/jamia/ocac019 PMC9006687

[29] Stiell, I. G. et al. The Canadian CT Head Rule for patients with minor head injury. *Lancet* **357**, 1391–1396 (2001). 11356436 10.1016/s0140-6736(00)04561-x

[30] Gianfrancesco, M. A., Tamang, S., Yazdany, J. & Schmajuk, G. Potential biases in machine learning algorithms using electronic health record data. *JAMA Intern. Med.* **178**, 1544–1547 (2018). 30128552 10.1001/jamainternmed.2018.3763 PMC6347576

[31] Heerink, J. S., Oudega, R., Hopstaken, R., Koffijberg, H. & Kusters, R. Clinical decision rules in primary care: necessary investments for sustainable healthcare. *Prim. Health Care Res. Dev.* **24**, e34 (2023). 37129072 10.1017/S146342362300021X PMC10156469

[32] Shapiro, S. E. Guidelines for developing and testing clinical decision rules. *West. J. Nurs. Res.* **28**, 244–253 (2006). 16513922 10.1177/0193945905283722

[33] Stiell, I. G. & Wells, G. A. Methodologic standards for the development of clinical decision rules in emergency medicine. *Ann. Emerg. Med.* **33**, 437–447 (1999). 10092723 10.1016/s0196-0644(99)70309-4

[34] Ebell, M. AHRQ White Paper: Use of clinical decision rules for point-of-care decision support. *Med. Decis. Mak. Int. J. Soc. Med. Decis. Mak.* **30**, 712–721 (2010). 10.1177/0272989X10386232 21183758

[35] Merone, L., Tsey, K., Russell, D. & Nagle, C. Sex inequalities in medical research: a systematic scoping review of the literature. *Women’s Health Rep.* **3**, 49 (2022). 10.1089/whr.2021.0083 PMC8812498 35136877

[36] Holdcroft, A. Gender bias in research: how does it affect evidence based medicine?. *J. R. Soc. Med.* **100**, 2 (2007). 17197669 10.1258/jrsm.100.1.2 PMC1761670

[37] Lee, E. & Wen, P. Gender and sex disparity in cancer trials. *ESMO Open* **5**, e000773 (2020). 32816862 10.1136/esmoopen-2020-000773 PMC7440710

[38] Poon, R. et al. Participation of women and sex analyses in late-phase clinical trials of new molecular entity drugs and biologics approved by the FDA in 2007–2009. *J. Women’s Health* **22**, 604 (2013). 10.1089/jwh.2012.3753 PMC3704049 23768021

[39] Geller, S. E. et al. The more things change, the more they stay the same: a study to evaluate compliance with inclusion and assessment of women and minorities in randomized controlled trials. *Acad. Med. J. Assoc. Am. Med. Coll.* **93**, 630–635 (2018). 10.1097/ACM.0000000000002027 PMC5908758 29053489

[40] Peiffer-Smadja, N. et al. Paving the Way for the Implementation of a Decision Support System for Antibiotic Prescribing in Primary Care in West Africa: Preimplementation and Co-Design Workshop With Physicians. *J. Med. Internet Res.* **22**, e17940 (2020). 32442155 10.2196/17940 PMC7400049

[41] Lovell, R. M. & Ford, A. C. Effect of gender on prevalence of irritable bowel syndrome in the community: systematic review and meta-analysis. *Am. J. Gastroenterol.* **107**, 991–1000 (2012). 22613905 10.1038/ajg.2012.131

[42] Gwee, K. A. et al. Second Asian Consensus on Irritable Bowel Syndrome. *J. Neurogastroenterol. Motil.* **25**, 343 (2019). 31327218 10.5056/jnm19041 PMC6657923

[43] Suriyakumar, V., Ghassemi, M. & Ustun, B. *When Personalization Harms: Reconsidering the Use of Group Attributes in Prediction*. 10.48550/arXiv.2206.02058 (2022).

[44] Eneanya, N. D., Yang, W. & Reese, P. P. Reconsidering the Consequences of Using Race to Estimate Kidney Function. *JAMA* **322**, 113–114 (2019). 31169890 10.1001/jama.2019.5774

[45] Grobman, W. A. et al. Prediction of vaginal birth after cesarean delivery in term gestations: a calculator without race and ethnicity. *Am. J. Obstet. Gynecol.* **225**, 664.e1–664.e7 (2021). 34043983 10.1016/j.ajog.2021.05.021 PMC8611105

[46] MDCalc Statement on Race. *MDCalc* https://www.mdcalc.com/race.

[47] Goodman, K. E., Blumenthal-Barby, J., Redberg, R. F. & Hoffmann, D. E. FAIRS - a framework for evaluating the inclusion of sex in clinical algorithms. *N. Engl. J. Med.* **392**, 404–411 (2025). 39778166 10.1056/NEJMms2411331

[48] Coots, M., Saghafian, S., Kent, D. M. & Goel, S. A framework for considering the value of race and ethnicity in estimating disease risk. *Ann. Intern. Med.* **178**, 98–107 (2025). 39622056 10.7326/M23-3166

[49] Six, A. J., Backus, B. E. & Kelder, J. C. Chest pain in the emergency room: value of the HEART score. *Neth. Heart J. Mon. J. Neth. Soc. Cardiol. Neth. Heart Found.* **16**, 191–196 (2008). 10.1007/BF03086144 PMC2442661 18665203

[50] Lyden, P. D. et al. A modified National Institutes of Health Stroke Scale for use in stroke clinical trials: preliminary reliability and validity. *Stroke* **32**, 1310–1317 (2001). 11387492 10.1161/01.str.32.6.1310

[51] Aromataris, E., Lockwood, C., Porritt, K., Pilla, B., Jordan, Z. *JBI Manual for Evidence Synthesis*. (JBI, 2024). 10.46658/JBIMES-24-01.

[52] Page, M. J. et al. The PRISMA 2020 statement: an updated guideline for reporting systematic reviews. *BMJ* **372**, n71 (2021). 33782057 10.1136/bmj.n71 PMC8005924

[53] Clayton, J. A. & Tannenbaum, C. Reporting Sex, Gender, or Both in Clinical Research? *JAMA* **316**, 1863–1864 (2016). 27802482 10.1001/jama.2016.16405

[54] Flanagin, A., Frey, T., Christiansen, S. L. & AMA Manual of Style Committee Updated Guidance on the Reporting of Race and Ethnicity in Medical and Science Journals. *JAMA* **326**, 621–627 (2021). 34402850 10.1001/jama.2021.13304

[55] Ouyang, L. et al. Training language models to follow instructions with human feedback. Preprint at 10.48550/arXiv.2203.02155 (2022).

