# Supplementary material for: Potential for Algorithmic Bias in Clinical Decision Instrument Development
Source: NPJ Digit Med. 2025 Dec 10;8:762. doi: 10.1038/s41746-025-02119-7 (PMC12708808; doi:10.1038/s41746-025-02119-7)
Supplement: Supplementary file 1 — Supplementary Information [file 41746_2025_2119_MOESM1_ESM.pdf]

Potential for Algorithmic Bias in Clinical Decision Instrument Development

Supplementary Information

Some predictor variables may be very frequent, but nevertheless relatively unimportant in CDIs. Fig 6 investigates the relative importance of frequent variables in CDIs. It analyzes scoring CDIs which assign points based on predictor variables; this excludes other types of CDIs, e.g. CDIs that use nonlinear formulas. We calculate variable importance as the fraction of total points. For example, if a CDI assigns 1 point for variable A and 3 points for variable B, then variable A receives a variable importance of ¼ and variable B receives a variable importance of ¾. Some very frequent variables (e.g. Sex, BMI) tend to have very low importance whereas some less frequent variables (e.g. C-reactive protein, BUN), tend to have higher importance.

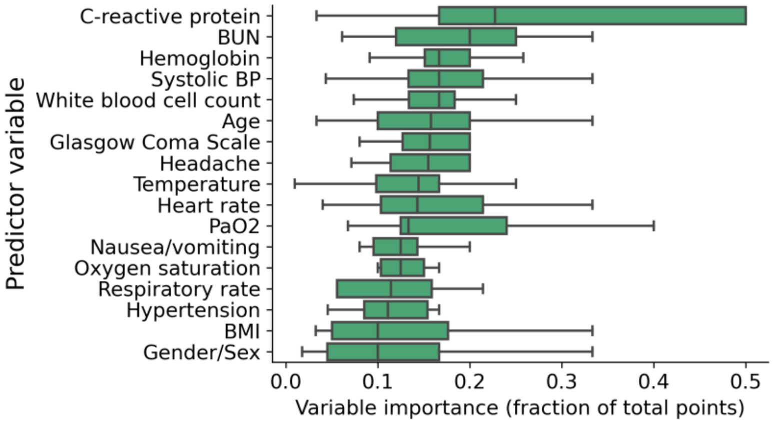

Supplementary Figure 1 | Variable importance of frequent predictor variables in CDIs. Restricted to scoring CDIS (309 / 690 CDIs) and to variables which appear in at least 9 scoring CDIs. Each box shows the median, interquartile range, and extended lines show 1.5 of the interquartile range. *Abbreviations. BP: Blood pressure, BMI: Body mass index, BUN: Blood urea nitrogen.*

Supplementary Table 1 | CDIs including *Race and Ethnicity* predictor variables. We characterize whether each variable is used in determining eligibility criteria for the CDI development cohort, in describing the likelihood of the outcome, or in modifying the risk of the outcome severity.

**CDI**	**Usage description**	**Race variable**	**Role of variable**
ASCVD (Atherosclerotic Cardiovascular Disease) 2013 Risk Calculator from AHA/ACC	10-year heart disease or stroke risk.	Race	Statistical predictor
ASCVD (Atherosclerotic Cardiovascular Disease) Risk Algorithm including Known ASCVD from AHA/ACC	10-year heart disease or stroke risk and statin recommendations.	Race	Statistical predictor
Australian Type 2 Diabetes Risk (AUSDRISK) Assessment Tool	Estimates diabetes risk.	Ethnicity	Statistical predictor
CKD-EPI Equations for Glomerular Filtration Rate (GFR)	Estimates GFR.	Race	Risk modifier
COVID-19 Inpatient Risk Calculator (CIRC)	Inpatient COVID mortality or severe disease progression.	White	Risk modifier
Denver HIV Risk Score	HIV risk screening.	Race and ethnicity	Statistical predictor
Estimated/Expected Peak Expiratory Flow (Peak Flow)	Quantifies asthma severity.	Race and ethnicity	Eligibility criteria
GWTG-Heart Failure Risk Score	Predicts in-hospital heart failure mortality.	Black race	Statistical predictor
Gail Model for Breast Cancer Risk	Breast CA risk based on demographic and	Race and ethnicity	Eligibility criteria

	clinical data.		
Kinetic Estimated Glomerular Filtration Rate (keGFR)	keGFR estimation.	Black race	Risk modifier
Licurse Score for Renal Ultrasound	Hydronephrosis probability.	Race	Statistical predictor
MDRD GFR Equation	For CKD patients (not for AKI).	Black race	Risk modifier
STONE Score for Uncomplicated Ureteral Stone	Predicts ureteral stones, before ultrasound.	Race	Statistical predictor

Supplementary Table 2 | CDIs including “Family history” variables.

CDI	Usage description	Family history variables	Role of Variable
ACC/AHA Heart Failure Staging	Heart failure staging and therapy recommendations.	Patient with family history of cardiomyopathy	Statistical predictor + risk modifier
ASAS Criteria for Axial Spondyloarthritis (SpA)	Axial SpA diagnosis.	Family history of SpA	Statistical predictor
ASAS Criteria for Peripheral SpondyloArthritis (SpA)	Peripheral SpA diagnosis.	Positive family history of SpA	Statistical predictor
CASPAR Criteria for Psoriatic Arthritis	Psoriatic arthritis diagnosis.	Current psoriasis, personal history of psoriasis, or family history of psoriasis	Statistical predictor
Cambridge Diabetes Risk Score	T2DM risk.	Family history	Statistical predictor
Caprini Score for Venous Thromboembolism (2005)	Stratifies risk of VTE in surgical patients.	Family history of thrombosis	Statistical predictor
Dutch Criteria for Familial Hypercholesterolemia (FH)	Diagnoses familial hypercholesterolemia.	Patient has elevated cholesterol, family history of FH, and/or family history of premature cardiac death	Statistical predictor
FINDRISC (Finnish Diabetes Risk Score)	T2DM risk.	Family history of diabetes	Statistical predictor
Opioid Risk Tool (ORT) for Narcotic Abuse	Risk of narcotic abuse/misuse.	Family history of illegal drug abuse, Family history of prescription drug abuse, Family history of alcohol abuse	Statistical predictor
Simon Broome Diagnostic Criteria for Familial Hypercholesterolemia (FH)	Diagnoses familial hypercholesterolemia (FH)	Family history of premature CVD events, Family history of extremely high cholesterol	Statistical predictor

Supplementary Table 3 | Renaming rules for predictor variables. Rules are applied starting from the left column: if a string contains a value in the *Case-insensitive match* column or the *Case-sensitive match* column, it is renamed to that value. Then, if a string starts with any of the prefixes in *Case-insensitive prefix match,* it is renamed to the prefix. Finally, any value matching a rule in *Case-sensitive rename* is renamed following the rule.

Case-insensitive match	Case-sensitive match	Case-insensitive prefix match	Case-sensitive rename
Ethnicity	ALT	Age	Systolic Blood Pressure → Systolic BP
D-dimer	AST	ASA	Ethnicity → Race and Ethnicity
Appetite	BMI	Albumin	White blood cell → White blood cell count
Level of consciousness	CRP	Anxiety	WBC → White blood cell count
Chest x-ray	C-reactive protein	Atrial fibrillation	CRP → C-reactive protein
Weight loss	CD4	ED visits	Diminished breath sounds → Decreased breath sounds
Karnofsky	CHF	EKG	EKG → ECG
Dysarthria	ECG	ESR	Female → Sex
Facial palsy	EKG	Endoscopy	GCS → Glasgow Coma Scale
Acidosis	HDL	BMI	Glasgow Coma Score → Glasgow Coma Scale
Abdominal pain	GCS	BUN	Pulse → Heart rate
Altered mental status	WBC	Biliary	Immobilization → Immobilized
Bilirubin	INR	Calcium	Intubation → Intubated
Eosinophilia	LDL	Congestive heart failure	Leukocyte → White blood cell count
Enthesitis	LDH	Creatinine	Vomiting → Nausea/vomiting
White blood cell	NIHSS	Dementia	NIHSS → NIH Stroke Scale
Erythrocyte sedimentation rate	NYHA	Distracting	Obesity → BMI
Estimated blood loss	PSA	ECOG	O_2 sat → Oxygen saturation
Female	$PaCO_2$	Erythema	PaO_2 → PaO2

Ferritin	PaO_2	Glucose	Patient age → Age
gestational age	PaO2	Hematocrit	Patient sex → Sex
Glasgow Coma Scale	sBP	Hemoglobin	Persistent vomiting → Nausea/vomiting
Heart rate		Race	Platelet → Platelet count
Pulse		Regional lymph node	Pregnant → Pregnancy
Headache		Respiratory rate	SpO_2 → Oxygen saturation
Height		Scalp hematoma	Sp02 → Oxygen saturation
Hematuria		Sex	Suicid → Suicidality
Fever		Systolic BP	Temp → Temperature
Blood in stool		Male	Tremor → Tremors
Diastolic BP		Nausea	Triglyceride → Triglycerides
Hypotension		Oxygen saturation	sBP → Systolic BP
Hypertension		Platelet	
Immobilized		Potassium	Diastolic blood pressure → Diastolic BP
Immobilization		Pregnancy	Systolic pressure → Systolic BP
Insulin		Pregnant	Nausea → Nausea/vomiting
Intraventricular hemorrhage		SaO_2	White → Race and Ethnicity
Intubation		Seizure	Race → Race and Ethnicity
Intubated		Sodium	Gender → Sex
Lactate		Sp02	Male → Sex
Length of stay		SpO_2	
Leukocyte		Temp	
Marked change in tone		Tremor	
NIH Stroke Scale		Triglyceride	
Age		Wheezing	
(Age		eGFR	
Verbal response		Weight	
Diastolic blood pressure		Suidicid	
Systolic pressure			
Vomiting			

Supplementary Table 4 | CDIs which have development cohorts that entirely consist of patients of a single sex. There are 13 male-only cohorts and 12 female-only cohorts. The mean fraction of male patients across all cohorts is 55.0%. When excluding single-sex cohorts, the mean fraction of male patients is 54.8%.

CDI	Description	# Male	# Female	Single-sex condition
CKD Prediction in HIV+ Patients	5 year likelihood of CKD in HIV.	21590	0	No
UCSF-CAPRA Score for Prostate Cancer Risk	Post-treatment outcomes for prostate cancer.	10627	0	Yes
Veterans Aging Cohort Study (VACS) 1.0 Index	All-cause mortality for patients with HIV or HCV.	4932	0	No
PSA Doubling Time (PSADT) Calculator	PSA survival.	1997	0	Yes
D'Amico Risk Classification for Prostate Cancer	Assesses 5 year failure of prostate CA treatment failure.	1872	0	Yes
Testicular Workup for Ischemia and Suspected Torsion (TWIST)	Testicular torsion diagnosis.	338	0	Yes
Bastion Classification of Lower Limb Blast Injuries	Lower limb blast injury.	103	0	No
Prostate Tumor Volume & Density	Tumor volume and PSA density.	100	0	Yes
Hamilton Depression Rating Scale (HAM-D)	Depression severity.	49	0	No
Pain Assessment in Advanced Dementia Scale (PAINAD)	Pain level in dementia patients.	19	0	No
CEDOCS Score for Emergency Department Overcrowding	Estimates severity of overcrowding in community EDs.	12	0	No
Phenytoin (Dilantin) Correction for Albumin / Renal Failure	Corrected phenytoin level.	9	0	No
Stanford Sleepiness Scale	Quantifies sleepiness.	5	0	No
Gail Model for Breast Cancer Risk	Breast CA risk based on demographic and clinical	0	284780	No

	data.			
Vaginal Birth After Cesarean (MFMU)	Successful vaginal birth prediction.	0	11687	Yes
VBAC Risk Score for Successful Vaginal Delivery (Flamm Model)	Quantifies likelihood of vaginal birth after previous C-section.	0	5022	Yes
Prevention and Incidence of Asthma and Mite Allergy (PIAMA) Risk Score	Risk of asthma for school age children.	0	4146	No
Modified Bishop Score for Vaginal Delivery and Induction of Labor	Likelihood of successful vaginal delivery.	0	1189	Yes
Charlson Comorbidity Index (CCI)	10-year survival.	0	685	No
Danger Assessment Tool for Domestic Abuse	Death by intimate partner violence.	0	666	No
BWH Egg Freezing Counseling Tool (EFCT)	Predicts likelihood of live birth for elective egg freezing in women.	0	520	Yes
Humiliation, Afraid, Rape, Kick (HARK)	Primary care domestic abuse detection.	0	232	No
Hurt, Insult, Threaten, Scream (HITS) Score	Detects domestic abuse in healthcare settings.	0	160	No
Risk of Malignancy Index (RMI) for Ovarian Cancer	Ovarian cancer risk for adnexal mass.	0	143	Yes
Bristol Stool Form Scale	Stool classification.	0	66	No

Supplementary Table 5 | Fraction of patient cohort identified as male, stratified by Disease, System, and Specialty. Categories containing less than 5 CDIs are excluded from this table. Some specialties have understandable imbalances, e.g. CDIs related to OB-Gyn have predominantly female patients whereas CDIs related to Urology have predominantly male patients.

Disease		System			Specialty	
Public Health	0.28	Reproductive		0.29	OB-Gyn	0.19
Rheumatoid Arthritis	0.34	Rheumatologic		0.36	Rheumatology	0.36
Depression/Suicidality	0.42	Any/All		0.47	Orthopedics	0.42
Pulmonary Embolism	0.45	Vascular		0.48	Oral and Maxillofacial Surgery	0.47
Diabetes Mellitus	0.47	Musculoskeletal		0.48	Allergy and Immunology	0.47
Deep Venous Thrombosis	0.47	Dermatologic		0.51	Radiation Oncology	0.49
Delirium	0.50	Psychiatric		0.52	Surgery (Vascular)	0.49
Sepsis	0.51	Respiratory		0.54	Psychiatry	0.50
Chronic Pain	0.51	Endocrine and Metabolic		0.55	Dermatology	0.51
Respiratory Failure	0.51	Cardiac		0.56	Family Practice	0.51
Anxiety	0.51	Hematologic		0.56	Endocrinology	0.52
Obesity	0.51	Neurologic		0.57	Neurosurgery	0.52
Congestive Heart Failure	0.52	Gastrointestinal		0.58	Pulmonology	0.53
Asthma	0.52	Oncologic		0.59	Primary Care	0.53
Appendicitis	0.52	Immunologic		0.59	Pediatrics	0.53
Autoimmune Disorders	0.53	Hepatic		0.65	Geriatrics	0.54
COPD	0.53	Infectious		0.68	Internal Medicine	0.54
Heart Failure	0.54	Renal		0.68	Surgery (General)	0.54
Endocarditis	0.54	Urinary		0.78	Hospitalist Medicine	0.54
Stroke/TIA	0.54				Anesthesiology	0.54
Chemotherapy	0.54				Critical Care	0.56
Atrial Fibrillation	0.54				Cardiology	0.56
Hypertension	0.55				Emergency Medicine	0.56
Respiratory Distress	0.55				Pain Management	0.56
COVID-19	0.56				Neurology	0.56
Arrhythmia	0.57				Surgery (Cardiothoracic)	0.57
Dementia	0.57				Pediatric Subspecialty	0.57
Coronary Artery Disease	0.57				Radiology	0.57
Pneumonia	0.58				Critical Care (Neurologic)	0.57
Hepatitis	0.58				Critical Care (Pediatric)	0.58
GI Bleeding	0.58				Gastroenterology	0.59
Cirrhosis	0.59				Hematology and Oncology	0.59
Hematologic Malignancy	0.59				Palliative Care/Hospice	0.59
Bleeding/Hemorrhage	0.59				Pharmacy	0.59
Cancer	0.60				Toxicology	0.59
Idiopathic Pulmonary Fibrosis	0.60				Hepatology	0.60
Myocardial Infarction	0.61				Rehabilitation Medicine	0.63
Acute Coronary Syndrome	0.61				Infectious Disease	0.63
Leukemia/Lymphoma	0.62				Surgery (Trauma)	0.63
Alcoholism	0.62				Nephrology	0.67
Anemia	0.64				Urology	0.83
Trauma	0.64					
Renal Failure	0.66					
Drug/Alcohol Use	0.67					
Hepatocellular Carcinoma	0.71					
HIV/AIDS	0.72					

Supplementary Table 6 | CDIs that involve follow-up.

Active	Hybrid	Passive
ABIC Score for Alcoholic Hepatitis	ARISCAT Score for Postoperative Pulmonary Complications	4-Level Pulmonary Embolism Clinical Probability Score (4PEPS)
AWOL Score for Delirium	Aortic Dissection Detection Risk Score (ADD-RS)	ACTION ICU Score for Intensive Care in NSTEMI
Acute Gout Diagnosis Rule	CHADS-VASc Score for Atrial Fibrillation Stroke Risk	ALBI (Albumin-Bilirubin) Grade for Hepatocellular Carcinoma (HCC)
Altitude-Adjusted PERC Rule	Canadian Transient Ischemic Attack (TIA) Score	ATLAS Score for Clostridium Difficile Infection
Asthma Predictive Index (API)	Charlson Comorbidity Index (CCI)	ATRIA Bleeding Risk Score
BODE Index for COPD Survival	Dual Antiplatelet Therapy (DAPT) Score	ATRIA Stroke Risk Score
Binet Staging System for Chronic Lymphocytic Leukemia (CLL)	Emergency Department Assessment of Chest Pain Score (EDACS)	Alberta Stroke Program Early CT Score (ASPECTS)
CATCH (Canadian Assessment of Tomography for Childhood Head injury) Rule	HAS-BLED Score for Major Bleeding Risk	Asymptomatic Myeloma Prognosis
Canadian C-Spine Rule	HEART Pathway for Early Discharge in Acute Chest Pain	Blast Lung Injury Severity Score
Canadian CT Head Injury/Trauma Rule	HEART Score for Major Cardiac Events	Brain Metastasis Velocity (BMV) Model
Canadian Syncope Risk Score	HIT Expert Probability (HEP) Score for Heparin-Induced Thrombocytopenia	CHADS₂ Score for Atrial Fibrillation Stroke Risk
Columbia Suicide Severity Rating Scale (C-SSRS Screener)	Indian Takayasu Clinical Activity Score (ITAS2010)	CKD Prediction in HIV+ Patients
D'Amico Risk Classification for Prostate Cancer	Modified Hoehn and Yahr Scale for Parkinson's Disease	CLIF-C ACLF (Acute-on-Chronic Liver Failure)
DIPSS (Dynamic International Prognostic Scoring System) for Myelofibrosis	PECARN Rule for Low Risk Febrile Infants 29-60 Days Old	COVID Home Safely Now (CHOSEN) Risk Score for COVID-19
DRAGON Score for Post-TPA Stroke Outcome	Simplified PESI (Pulmonary Embolism Severity Index)	COVID-19 Inpatient Risk Calculator (CIRC)
Danger Assessment Tool for Domestic Abuse	Vancouver Chest Pain Rule	Clinical Disease Activity Index (CDAI) for Rheumatoid Arthritis
Disease Activity Score-28 for Rheumatoid Arthritis with CRP (DAS28-CRP)	Wells' Criteria for Pulmonary Embolism	DASH Prediction Score for Recurrent VTE
Disease Activity Score-28 for Rheumatoid Arthritis with ESR (DAS28-ESR)		DIRE Score for Opioid Treatment
Disease Steps for Multiple Sclerosis		Duval/CIBMTR Score for Acute Myelogenous Leukemia (AML) Survival
Duke Treadmill Score		EUTOS Score for Chronic Myelogenous Leukemia (CML)
EGSYS (Evaluation of Guidelines in SYncope Study) Score for Syncope		European System for Cardiac Operative Risk Evaluation (EuroSCORE) II
Endotracheal Tube (ETT) Depth and Tidal Volume Calculator		FINDRISC (Finnish Diabetes Risk Score)
Fong Clinical Risk Score for Colorectal Cancer Recurrence		Fibrosis-4 (FIB-4) Index for Liver Fibrosis
Functional Outcome in Patients With Primary Intracerebral Hemorrhage (FUNC) Score		Follicular Lymphoma International Prognostic Index (FLIPI)
Geneva Score (Revised) for Pulmonary Embolism		For Patients: VACO Index COVID-19 Mortality Risk
Groupe d'Etude des Lymphomes Folliculaires (GELF) Criteria		Fuhrman Nuclear Grade for Clear Cell Renal Carcinoma
HERDOO2 Rule for Discontinuing Anticoagulation in Unprovoked VTE		GALAD Model for Hepatocellular Carcinoma (HCC)
Hestia Criteria for Outpatient Pulmonary Embolism Treatment		GAP Index for Idiopathic Pulmonary Fibrosis (IPF) Mortality
LACE Index for Readmission		GRACE ACS Risk and Mortality Calculator
Lille Model for Alcoholic Hepatitis		Gail Model for Breast Cancer Risk

Liver Decompensation Risk after Hepatectomy for Hepatocellular Carcinoma (HCC)		Glasgow Prognostic Score (GPS) for Cancer Outcomes
Marburg Heart Score (MHS)		Gupta Postoperative Respiratory Failure Risk
Modified Asthma Predictive Index (mAPI)		HAT (Hemorrhage After Thrombolysis) Score for Predicting Post-tPA Hemorrhage
NEXUS Chest Decision Instrument for Blunt Chest Trauma		HEMORR₂HAGES Score for Major Bleeding Risk
Ocular Hypertension Treatment Study (OHTS) Calculator		HINTS for Stroke in Acute Vestibular Syndrome
Ottawa Subarachnoid Hemorrhage (SAH) Rule for Headache Evaluation		HOSPITAL Score for Readmissions
PECARN Pediatric Head Injury/Trauma Algorithm		HScore for Reactive Hemophagocytic Syndrome
Padua Prediction Score for Risk of VTE		Hour-Specific Risk for Neonatal Hyperbilirubinemia
Palchak (UC Davis) Rule for Pediatric Head Trauma		Hypoglycemia Risk Score
Pediatric Asthma Severity Score (PASS) for Asthma Exacerbation Severity		IMDC (International Metastatic RCC Database Consortium) Risk Model for Metastatic Renal Cell Carcinoma
Pediatric NEXUS II Head CT Decision Instrument for Blunt Trauma		IMPACT Score for Outcomes in Head Injury
Prevention and Incidence of Asthma and Mite Allergy (PIAMA) Risk Score		IMPROVE RAM (International Medical Prevention Registry on Venous Thromboembolism Risk Assessment Model)
Psoriasis Area and Severity Index (PASI)		IMPROVE Risk Score for Venous Thromboembolism (VTE)
Revised Natural History Model for Primary Sclerosing Cholangitis		IMPROVEDD Risk Score for Venous Thromboembolism (VTE)
STONE Nephrolithometry Score for Renal Calculi		Infective Endocarditis (IE) Mortality Risk Score
Simple Disease Activity Index (SDAI) for Rheumatoid Arthritis		International Prognostic Index for Chronic Lymphocytic Leukemia (CLL-IPI)
Steinhart Model for Acute Heart Failure (AHF) in Undifferentiated Dyspnea		Khorana Risk Score for Venous Thromboembolism in Cancer Patients
Systemic Lupus Erythematosus Disease Activity Index 2000 (SLEDAI-2K)		Kidney Failure Risk Calculator
Troponin-only Manchester Acute Coronary Syndromes (T-MACS) Decision Aid		Killip Classification for Heart Failure
Truelove and Witts Severity Index for Ulcerative Colitis		Kruis Score for Diagnosis of Irritable Bowel Syndrome (IBS)
Urticaria Activity Score (UAS)		LENT Prognostic Score for Malignant Pleural Effusion
WPSS (WHO classification-based Prognostic Scoring System) for Myelodysplastic Syndrome		Leiden Clinical Prediction Rule for Undifferentiated Arthritis
YEARS Algorithm for Pulmonary Embolism (PE)		MAGGIC Risk Calculator for Heart Failure
		MELD Na (UNOS/OPTN)
		MELD Score (Original, Pre-2016, Model for End-Stage Liver Disease)
		Mantle Cell Lymphoma International Prognostic Index (MIPI)
		Mekhail Extension of the Motzer Score
		Memorial Sloan-Kettering Cancer Center (MSKCC/Motzer) Score for Metastatic Renal Cell Carcinoma (RCC)
		Metroticket Calculator for Hepatocellular Carcinoma (HCC) Survival
		Michigan Risk Score for PICC-Related Thrombosis

		Mirels’ Criteria for Prophylactic Fixation
		Modified Glasgow Prognostic Score (mGPS) for Cancer Outcomes
		Modified NIH Stroke Scale/Score (mNIHSS)
		Montreal Classification for Inflammatory Bowel Disease (IBD)
		Morphine Milligram Equivalents (MME) Calculator
		Mumtaz Score for Readmission in Cirrhosis
		NEXUS Chest CT Decision Instrument for CT Imaging
		Nutritional Risk Index (NRI)
		ORBIT Bleeding Risk Score for Atrial Fibrillation
		Opioid Risk Tool (ORT) for Narcotic Abuse
		Ottawa COPD Risk Scale
		Ottawa Heart Failure Risk Scale (OHFRS)
		PEDIS Score for Diabetic Foot Ulcers
		PLASMIC Score for TTP
		POMPE-C Tool for Pulmonary Embolism Mortality
		POSSUM for Operative Morbidity and Mortality Risk
		PREVAIL Model for Prostate Cancer Survival
		PRIEST COVID-19 Clinical Severity Score
		PSA Doubling Time (PSADT) Calculator
		Patient Activity Scale (PAS) for RA
		Patient Activity Scale II (PAS II) for RA
		Pittsburgh Response to Endovascular therapy (PRE) Score
		Preoperative Mortality Predictor (PMP) Score
		Prognostic Index for Cancer Outcomes
		Pulmonary Embolism Severity Index (PESI)
		RCVS₂ Score for Reversible Cerebral Vasoconstriction Syndrome
		REACH-B Score for Hepatocellular Carcinoma (HCC)
		READMITS Score for Readmissions in Acute MI
		REVEAL Registry Risk Score 2.0 for Pulmonary Arterial Hypertension (PAH)
		ROSE (Risk Stratification of Syncope in the Emergency Department) Rule
		Rai Staging System for Chronic Lymphocytic Leukemia (CLL)
		Recurrent Instability of the Patella (RIP) Score
		RegiSCAR Score for Drug Reaction with Eosinophilia and Systemic Symptoms (DRESS)
		Revised International Prognostic Scoring System (IPSS-R) for Myelodysplastic Syndrome (MDS)
		Revised Multiple Myeloma International Staging System (R-ISS)
		Reynolds Risk Score for Cardiovascular Risk in Women
		Risk of Paradoxical Embolism (RoPE) Score

		Salivary Gland Cancer Model for Survival +/- Postoperative Radiotherapy (PORT)
		Simon Broome Diagnostic Criteria for Familial Hypercholesterolemia (FH)
		Sokal Index for Chronic Myelogenous Leukemia (CML)
		THRIVE Score for Stroke Outcome
		Therapy-Disability-Neurology (TDN) Grade
		UCLA Integrated Staging System (UISS) for Renal Cell Carcinoma (RCC)
		UCSF-CAPRA Score for Prostate Cancer Risk
		United Kingdom Model for End-Stage Liver Disease (UKELD)
		Veterans Aging Cohort Study (VACS) 1.0 Index
		Veterans Health Administration COVID-19 (VACO) Index for COVID-19 Mortality

Supplementary Table 7 | CDIs without retrievable primary literature

CDI Name	Description
Bicarbonate Deficit	Calculates total body bicarb deficit.
LDL Calculated	From Total Chol, HDL and Trigs.
Pregnancy Due Dates Calculator	From LMP, EGA, or date of conception.
Steroid Conversion Calculator	Steroid dosing equivalencies.
Gleason Score for Prostate Cancer	Prognoses prostate cancer.
Immunization Schedule Calculator	Immunizations due based on age.
Schwab and England Activities of Daily Living (ADL) Scale	Parkinson's disease disability.
New York Heart Association (NYHA) Functional Classification for Heart Failure	Stratifies severity of heart failure.
DSM-5 Criteria for Binge Eating Disorder	Diagnosis of BED.
Pack Years Calculator	Quantify smoking history.
DSM-5 Criteria for Major Depressive Disorder	Diagnosis of MDD.
DSM-5 Criteria for Bipolar Disorder	Diagnosis of BPD.
DSM-5 Criteria for Posttraumatic Stress Disorder	Diagnosis of PTSD.
Utah COVID-19 Risk Score	Oral antiviral recommendation.
Indications for Paxlovid	Paxlovid indication.
Revised Urinary Incontinence Scale (RUIS)	Assesses for incontinence.
Urine Output and Fluid Balance	Urine output over 24 hrs.
Atropine Dosing for Cholinesterase Inhibitor Toxicity	Cholinesterase inhibitor reversal.
Karnofsky Performance Status Scale	Chemotherapy tolerance.
Estimated Ethanol (and Toxic Alcohol) Serum Concentration Based on Ingestion	Predicts ethanol concentration based on ingestion of alcohol.
Dutch Criteria for Familial Hypercholesterolemia (FH)	Diagnoses familial hypercholesterolemia.
Global Initiative for Obstructive Lung Disease (GOLD) Criteria for COPD	COPD staging and recommendations.
High-dose Insulin Euglycemia Therapy (HIET)	Calcium-channel blocker, beta blocker reversal.
Fomepizole Dosing	Methanol and ethylene glycol reversal.
Sickle Cell RBC Exchange Volume	Donor RBC volume for exchange.
Cryoprecipitate Dosing for Fibrinogen Replacement	Cryoprecipitate dosing.
Maternal-Fetal Hemorrhage Rh(D) Immune Globulin Dosage	RhIG for HDFN.
RBC Units to Screen for Compatibility	Donors to antigen type.
Donor Lymphocyte Infusion (DLI) Volume	Estimates DLI dosage.
Modified Finnegan Neonatal Abstinence Score (NAS)	Opioid withdrawal in newborns.

Visual Acuity Testing (Snellen Chart)	Visual acuity.
Color Vision Screening (Ishihara Test)	Color blindness.
Benzodiazepine Conversion Calculator	Benzodiazepine conversion.
Cardiac Output (Fick's Formula)	Calculates CO, CI, and SV.
Villalta Score for Post-thrombotic Syndrome (PTS)	Post-thrombotic syndrome severity.
Local Anesthetic Dosing Calculator	Local anesthetic toxicity prevention.
Brescia-COVID Respiratory Severity Scale (BCRSS)/Algorithm	COVID-19 pathway/approach from Italy.
ACEP ED COVID-19 Management Tool	ED management tool for COVID-19.

Supplementary Table 8 | PRISMA 2020 Checklist and Abstract Checklist

PRISMA 2020 Main Checklist

Topic	No.	Item	Location where item is reported
**TITLE**			
**Title**	1	Identify the report as a systematic review.	Title
**ABSTRACT**			
**Abstract**	2	See the PRISMA 2020 for Abstracts checklist	
**INTRODUCTION**			
**Rationale**	3	Describe the rationale for the review in the context of existing knowledge.	Introduction Paragraph 1
**Objectives**	4	Provide an explicit statement of the objective(s) or question(s) the review addresses.	Introduction Paragraph 3
**METHODS**			
**Eligibility criteria**	5	Specify the inclusion and exclusion criteria for the review and how studies were grouped for the syntheses.	Methods, Paragraph 1
**Information sources**	6	Specify all databases, registers, websites, organisations, reference lists and other sources searched or consulted to identify studies. Specify the date when each source was last searched or consulted.	Methods, Paragraph 1
**Search strategy**	7	Present the full search strategies for all databases, registers and websites, including any filters and limits used.	Methods, Paragraph 1
**Selection process**	8	Specify the methods used to decide whether a study met the inclusion criteria of the review, including how many reviewers screened each record and each report retrieved, whether they worked independently, and if applicable, details of automation tools used in the process.	Methods, Paragraph 1

Topic	No.	Item	Location where item is reported
**Data collection process**	9	Specify the methods used to collect data from reports, including how many reviewers collected data from each report, whether they worked independently, any processes for obtaining or confirming data from study investigators, and if applicable, details of automation tools used in the process.	Methods, Paragraph 1-4, 7
**Data items**	10a	List and define all outcomes for which data were sought. Specify whether all results that were compatible with each outcome domain in each study were sought (e.g. for all measures, time points, analyses), and if not, the methods used to decide which results to collect.	Methods, Paragraph 2-6
	10b	List and define all other variables for which data were sought (e.g. participant and intervention characteristics, funding sources). Describe any assumptions made about any missing or unclear information.	Methods, Paragraph 2-6
**Study risk of bias assessment**	11	Specify the methods used to assess risk of bias in the included studies, including details of the tool(s) used, how many reviewers assessed each study and whether they worked independently, and if applicable, details of automation tools used in the process.	Methods, Paragraph 1,5,7
**Effect measures**	12	Specify for each outcome the effect measure(s) (e.g. risk ratio, mean difference) used in the synthesis or presentation of results.	N/A
**Synthesis methods**	13a	Describe the processes used to decide which studies were eligible for each synthesis (e.g. tabulating the study intervention characteristics and comparing against the planned groups for each synthesis (item 5)).	Methods, Paragraph 1
	13b	Describe any methods required to prepare the data for presentation or synthesis, such as handling of missing summary statistics, or data conversions.	Methods, Paragraph 2-3, 5; Results, Paragraph 3
	13c	Describe any methods used to tabulate or visually display results of individual studies and syntheses.	Results, Table 1, Figure 1-3
	13d	Describe any methods used to synthesize results and provide a rationale for the choice(s). If meta-analysis was performed, describe the model(s), method(s) to identify the presence and extent of statistical heterogeneity, and software package(s) used.	N/A

Topic	No.	Item	Location where item is reported
	13e	Describe any methods used to explore possible causes of heterogeneity among study results (e.g. subgroup analysis, meta-regression).	Methods, Paragraph 2-3, 5
	13f	Describe any sensitivity analyses conducted to assess robustness of the synthesized results.	Methods, Paragraph 3
**Reporting bias assessment**	14	Describe any methods used to assess risk of bias due to missing results in a synthesis (arising from reporting biases).	Methods, Paragraph 1-2
**Certainty assessment**	15	Describe any methods used to assess certainty (or confidence) in the body of evidence for an outcome.	Methods, Paragraph 7
**RESULTS**			
**Study selection**	16a	Describe the results of the search and selection process, from the number of records identified in the search to the number of studies included in the review, ideally using a flow diagram.	Results, Paragraph 1
	16b	Cite studies that might appear to meet the inclusion criteria, but which were excluded, and explain why they were excluded.	Results, Paragraph 2; Supplementary Table 7
**Study characteristics**	17	Cite each included study and present its characteristics.	Results, Paragraph 1
**Risk of bias in studies**	18	Present assessments of risk of bias for each included study.	Results, Discussion
**Results of individual studies**	19	For all outcomes, present, for each study: (a) summary statistics for each group (where appropriate) and (b) an effect estimate and its precision (e.g. confidence/credible interval), ideally using structured tables or plots.	Results
**Results of syntheses**	20a	For each synthesis, briefly summarise the characteristics and risk of bias among contributing studies.	Results, Paragraph 1, 3-4, 5, 7-8
	20b	Present results of all statistical syntheses conducted. If meta-analysis was done, present for each the summary estimate and its precision (e.g. confidence/credible interval) and measures of statistical heterogeneity. If comparing groups, describe the direction of the effect.	Results, Paragraph 1, 3-4, 5, 7-8
	20c	Present results of all investigations of possible causes of heterogeneity among study results.	Results, Paragraph 1, 3-4, 5, 7-8

Topic	No.	Item	Location where item is reported
	20d	Present results of all sensitivity analyses conducted to assess the robustness of the synthesized results.	Results, Paragraph 1, 3-4, 5, 7-8
**Reporting biases**	21	Present assessments of risk of bias due to missing results (arising from reporting biases) for each synthesis assessed.	Results Paragraph 2, Figure 1, Figure 2
**Certainty of evidence**	22	Present assessments of certainty (or confidence) in the body of evidence for each outcome assessed.	Results, Paragraph 1-2
**DISCUSSION**			
**Discussion**	23a	Provide a general interpretation of the results in the context of other evidence.	Discussion, Paragraph 2
	23b	Discuss any limitations of the evidence included in the review.	Discussion, Paragraph 6
	23c	Discuss any limitations of the review processes used.	Discussion, Paragraph 6
	23d	Discuss implications of the results for practice, policy, and future research.	Discussion, Paragraph 2-5
**OTHER INFORMATION**			
**Registration and protocol**	24a	Provide registration information for the review, including register name and registration number, or state that the review was not registered.	N/A
	24b	Indicate where the review protocol can be accessed, or state that a protocol was not prepared.	N/A
	24c	Describe and explain any amendments to information provided at registration or in the protocol.	N/A
**Support**	25	Describe sources of financial or non-financial support for the review, and the role of the funders or sponsors in the review.	Acknowledgements
**Competing interests**	26	Declare any competing interests of review authors.	Competing Interests
**Availability of data, code and other materials**	27	Report which of the following are publicly available and where they can be found: template data collection forms; data extracted from included studies; data used for all analyses; analytic code; any other materials used in the review.	Data Availability

PRIMSA Abstract Checklist

Topic	No.	Item	Reported?
**TITLE**			
**Title**	1	Identify the report as a systematic review.	Yes
**BACKGROUND**			
**Objectives**	2	Provide an explicit statement of the main objective(s) or question(s) the review addresses.	Yes
**METHODS**			
**Eligibility criteria**	3	Specify the inclusion and exclusion criteria for the review.	No
**Information sources**	4	Specify the information sources (e.g. databases, registers) used to identify studies and the date when each was last searched.	No
**Risk of bias**	5	Specify the methods used to assess risk of bias in the included studies.	Yes
**Synthesis of results**	6	Specify the methods used to present and synthesize results.	Yes
**RESULTS**			
**Included studies**	7	Give the total number of included studies and participants and summarise relevant characteristics of studies.	Yes
**Synthesis of results**	8	Present results for main outcomes, preferably indicating the number of included studies and participants for each. If meta-analysis was done, report the summary estimate and confidence/credible interval. If comparing groups, indicate the direction of the effect (i.e. which group is favoured).	Yes
**DISCUSSION**			
**Limitations of evidence**	9	Provide a brief summary of the limitations of the evidence included in the review (e.g. study risk of bias, inconsistency and imprecision).	No
**Interpretation**	10	Provide a general interpretation of the results and important implications.	Yes
**OTHER**			
**Funding**	11	Specify the primary source of funding for the review.	No
**Registration**	12	Provide the register name and registration number.	No
